# Comparative Transcriptomic Analysis of Grape Berry in Response to Root Restriction during Developmental Stages

**DOI:** 10.3390/molecules21111431

**Published:** 2016-10-28

**Authors:** Feng Leng, Qiong Lin, Di Wu, Shiping Wang, Dengliang Wang, Chongde Sun

**Affiliations:** 1 Laboratory of Fruit Quality Biology, The State Agriculture Ministry Laboratory of Horticultural Plant Growth, Development and Quality Improvement, Zhejiang University, Zijingang Campus, Hangzhou 310058, China; lengfeng.214@163.com (F.L.); linqiong@zju.edu.cn (Q.L.); adesun2006@zju.edu.cn (D.W.); 2 Institute of Agro-Food Science and Technology, Chinese Academy of Agricultural Sciences/Key Opening Laboratory of Agricultural Products Processing and Quality Control, Ministry of Agriculture, Beijing 100193, China; 3 School of Agriculture and Biology, Shanghai Jiao Tong University, Shanghai 200240, China; fruit@sjtu.edu.cn; 4 Quzhou Academy of Agricultural Science, Quzhou 324000, China; dengliangwang001@163.com

**Keywords:** grape berry, root restriction, RNA-Seq, transcriptome

## Abstract

Root restriction improved berry quality by being involved in diverse aspects of grapevine life. However, the molecular mechanism driving this process is not understood very well. In this study, the ‘Summer Black’ grape berry (*Vitis vinifera × V. labrusca*) under root restriction was investigated, which showed an increase of total soluble solids (TSS), color index of red grapes (CIRG) value, anthocyanins accumulation, total phenolics and total procyanidins contents during berry development compared with those in control berries. The transcriptomic changes induced by root restriction in ‘Summer Black’ grape over the course of berry development were analyzed by RNA-Seq method. A total of 29,971 genes were generated in ‘Summer Black’ grape berry during development, among which, 1606 genes were significantly responded to root restriction. Furthermore, 1264, 313, 141, 246 and 19 sequences were significantly changed at S1, S2, S3, S4 and S5 sample points, respectively. The gene (*VIT_04s0023g02290*) predicted as a salicylate *O*-methyltransferase was differentially expressed in all developmental stages. Gene Ontology (GO) enrichment showed that response to organic nitrogen, response to endogenous stimulus, flavonoid metabolic process, phenylpropanoid biosynthetic process and cell wall macromolecule metabolic process were the main significant differential categories. Kyoto Encyclopedia of Genes and Genomes (KEGG) pathway enrichment revealed plant–pathogen interaction, plant hormone signal transduction, flavone and flavonol biosynthesis, flavonoid biosynthesis and glucosinolate biosynthesis were the main significant differential pathways. The results of the present study provided a genetic base for the understanding of grape berry fruit quality improvement under root restriction.

## 1. Introduction

Grapes are an important economical fruit species worldwide [1]. As a non-climacteric fruit, it follows a double sigmoidal growth curve with three major phases [2]. Each phase undergoes a complex series of changes on color, metabolic composition and gene expression [3].

Grapes are sensitive to root zone stresses such as water limit and salinity [4,5,6,7]. In addition, root restriction (RR) is another type of stress for grape cultivation based on the restriction of roots in a limited volume by physical or ecological methods [8,9]. Previous reports demonstrated that grapevines subjected to RR displayed different growth habits compared with those under normal cultivation. For example, RR limited the shoots and roots growth, enhanced the nitrate uptake rate and improved the fruit quality [8,10,11,12]. Some research also indicated that RR significantly increased the total sugar content, and the total and individual anthocyanin levels, which were in conformity with the upregulated expression of related genes [9,13,14]. 

The draft whole-genome sequence of the Pinot Noir grapevine obtained by Jaillon et al. [15] provides a novel, high-throughput, deep-sequenced, more insightful and accurate method to analyze the functional complexity of transcriptomes [16,17,18]. Recently, developed RNA-Seq provides a more comprehensive approach to study transcripts’ functional categories and their secondary metabolites [19]. Although several transcriptome studies were performed using RNA-Seq in grape berry during developmental stages [19,20,21,22] and under stresses [6,7,23], no comparative transcriptome analysis influenced by RR during berry development was complemented yet.

In the present study, the Illumina RNA-Seq method was carried out to identify and analyze the transcriptome changes in grape berry treated by RR during berry development. On the basis of comparing and analyzing the regulation difference between control and RR-treated berries, this paper provided a genetic resource for fruit quality improvement study.

## 2. Results and Discussion

### 2.1. Physical Properties of Grapes

**Figure 1:**
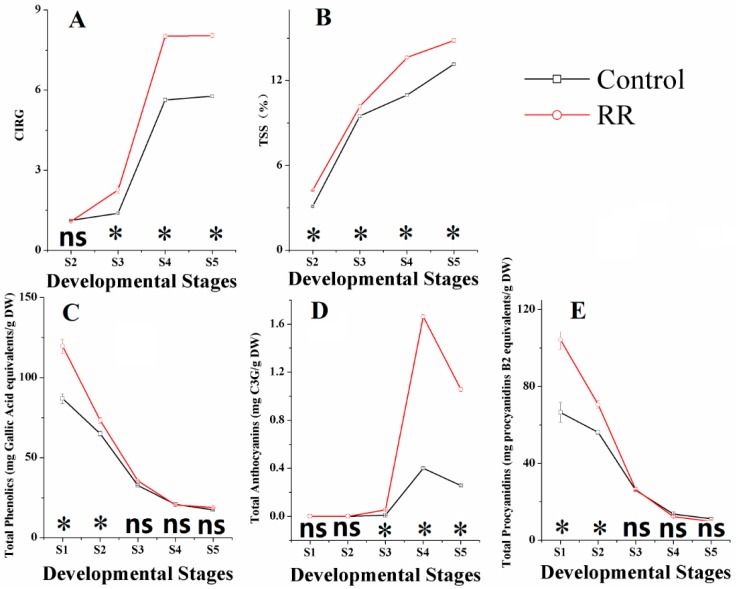
Effects of root restriction on berry parameters in different developmental stages. (**A**) chromatic aberration; (**B**) total soluble solids; (**C**) total phenolics; (**D**) total anthocyanins; (**E**) total procyanidins. S1, fruitlet; S2, immature green; S3, before veraison; S4, veraison; S5, fully ripe. * indicates significant differences (*p* < 0.05), ns = not significant differences (*p* > 0.05).

Different physical properties, including chromatic aberration, total soluble solids (TSSs), total phenolics, total anthocyanins and total procyanidins, were measured throughout the development in ‘Summer Black’ berries with both control and RR treatments. Results showed that color index of red grapes (CIRG) value and TSS significantly increased in RR treated berries and had similar tendencies for both treatments during the whole sampling period. CIRG value increased steadily before veraison, and then sharply ascended at the veraison period, reaching approximately 14 and nine for the RR treatment and control at the fully ripe stage, respectively. TSS increased rapidly at the immature green period and then became steady, reaching 14.6 and 13.6 brix for RR and control at the fully ripe stage, respectively (Figure 1A,B). These results were well in accordance with previous studies [9,24].

Compared with total phenolics and total procyanidins of berries, total anthocyanins were influenced much more intensively by RR (Figure 1C–E). Anthocyanins are important secondary metabolites and usually stored as glycosylated forms in vacuoles [25]. Anthocyanin accumulation starts at the onset of veraison and reaches the maximum around harvest time, and then there is a slight decrease at harvest and during over-ripening periods [26,27,28,29]. From our results, RR accumulation of anthocyanins started earlier and significantly increased the total concentration at the veraison and fourfold the amount of the control at the fully ripe stage. Similar results were also obtained in previous works [13,14]. Total phenolics and total procyanidins in both treatments decreased continually and shared similar trends during the entire sampling period in our experiments, which was basically consistent with previous results [30]. RR treatment obtained higher concentration of the total phenolics and lower concentration of the total procyanidins than the control at harvest. Phenolics compounds, such as flavonols, resveratrols, procyanidins and anthocyanins have a common synthetic pathway and can be induced by external stimuli [23]. Procyanidins are flavan-3-ol oligomers, concentration and the degree of polymerization changes occurring during berry development are complex [7], thus the mechanisms of the total phenolics, total anthocyanins and total procyanidins influenced by RR needs further research.

### 2.2. Evaluation of RNA Sequencing Data

To obtain a global view of the transcriptome of grape berries, high-throughput RNA-Seq using Illumina Hiseq 2000 sequencing platform (Majorbio Biopharm Technology Co., Ltd., Shanghai, China) was performed on RNAs during the developmental stages for both treatments. RNA-Seq analysis generated about 150 Gb of sequence data, and every sample was represented by over 40 million reads, which is enough for the quantitative analysis of gene expression. All of the raw and clean data and their qualities are listed in Table 1. The raw reads were trimmed by removing low-quality reads and adapters. The Q30 scores of clean bases were approximately 93% for these samples. The quality was assessed by saturation analysis. Duplicate reads analysis and gene coverage analysis indicated the RNA-Seq data was suitable for subsequent analyses (data are not shown). The sequence reads were then matched to the grape reference genome database by TopHat 2.0.13 software (http://tophat.cbcb.umd.edu/). The mapped ratio was about 60%–70% (Table 1), which suggested that it was probably generated from alternative splicing, new transcripts, cultivation environment or different varieties compared with the reference *Vitis vinifera* genome. 

### 2.3. Differential Gene Expression Triggered by Root Restriction

After aligning and assembling, the expression of 29,971 genes was detected for both treatments during the berry development by removal of partial overlapping sequences. Their expressions in five developmental stages of two treatments were summarized in Table 2. Among these genes with a total of 1606 significant differential expressions, 1264 (987 upregulated and 277 downregulated), 313 (72 upregulated and 241 downregulated), 141 (112 upregulated and 29 downregulated), 246 (158 upregulated and 88 downregulated) and 19 (11 upregulated and 8 downregulated) sequences were significantly changed at least two-fold in RR treatment in S1, S2, S3, S4 and S5, respectively, compared with controls. A total of 321 genes were detected to be expressed at more than one developmental stage, but only one gene (*VIT_04s0023g02290*) had differential significantly expression in all development stages, which was predicted to be a salicylate *O*-methyltransferase gene. These results indicated that many genes responded positively to RR treatment, which was similar to the previous report [31].

**Figure 2:**
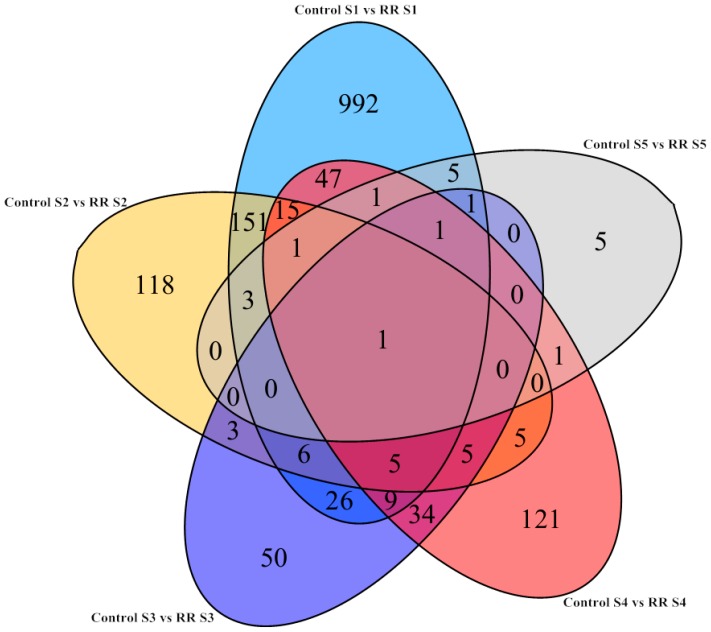
Venn diagrams showing the number of overlapping and non-overlapping genes with significantly differential expression levels (FDR < 0.05 and Log_2_FC > 1) during developmental stages under root restriction treatment. Ligte blue, the number of genes at the fruitlet stage; Yellow, the number of genes at the immature green stage; Dark blue, the number of genes at the before veraison stage; Red, the number of genes at the veraison stage; Gray, the number of genes at the fully ripe stage. FDR, false discovery rate; FC, fold change.

All of the differentially expressed genes during berry development were visualized by a Venn diagram. There were a relatively large number of these genes that were specifically upregulated and downregulated at fruitlet periods. Therefore, the differentially expressed genes between young and fully ripe berries were used for further research (Figure 2). Many genes with high expression for young berries could be linked with the photosynthetic capacity at the early stages of development, which decreased significantly during ripening [32].

### 2.4. GO Functional Annotation and KEGG Analysis

**Figure 3:**
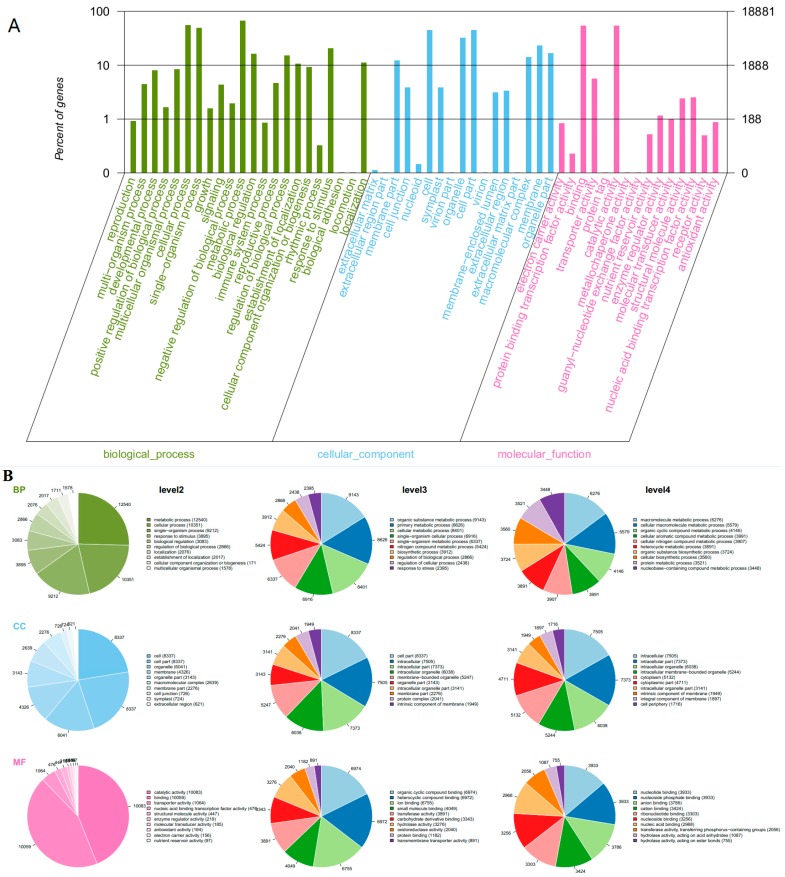
Gene Ontology (GO) functional annotation of genes detected in grape berries. (**A**) the **right** *y*-axis represents the number of genes in a sub-category. The **left** *y*-axis indicates the percentage of a specific sub-category of genes in each main category; (**B**) classification of GO terms. BP, biological process; CC, cellular component; MF, molecular function.

Gene Ontology (GO) as an international standardized gene functional analyses system was used to classify the functions of the transcripts during grape development [33]. Some genes were annotated with three main functional categories: a gene might be active in one or more biological processes, associated with or located in one or more cellular components, performed one or more molecular functions. Across all the samples, 18,881 transcripts (63%) were categorized into 54 functional groups based on sequence homology (Figure 3A). In three main categories (biological process, cellular component, molecular function) of the GO classification, there were 22, 17 and 15 functional groups, respectively. “Metabolic process” (GO: 0008152, 12540 transcripts), “cellular process” (GO: 0009987, 10351 transcripts) and “single-organism process” (GO: 0044699, 9212 transcripts) were predominant for biological processes. In the cellular component, the three main groups were “cell” (GO: 0005623, 8337 transcripts), “cell part” (GO: 0044464, 8337 transcripts) and “organelle” (GO: 0043226, 6041 transcripts). In the category of molecular function, “catalytic activity” (GO: 0003824, 10083 transcripts), “binding” (GO: 0005488, 10059 transcripts) and “transporter activity” (GO: 0005215, 1064 transcripts) were the most common groups (Figure 3B). GO enrichment analysis revealed statistically significant differences of functional categories between two treatments during these developmental stages (Tables S1–S5 in Supplemental file 1). It was noticed that there was a high ratio of differential genes from functional groups of biological process throughout all developmental stages induced by root restriction.

**Figure 4:**
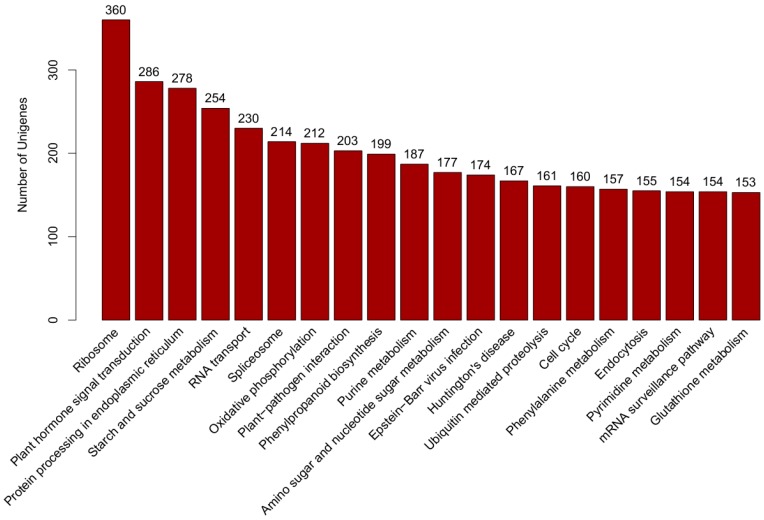
Kyoto Encyclopedia of Genes and Genomes (KEGG) pathway.

Kyoto Encyclopedia of Genes and Genomes (KEGG) pathway annotation is a useful tool to understand the biological functions of genes. According to the knowledge base for systematic analysis of genomic and functional information, whose results were retrieved from KEGG database according to sequence similarity [34], there were 9556 transcripts assigned to 325 KEGG pathways. The top five main pathways were “ribosome” (ko03010, 360 transcripts), “plant hormone signal transduction” (ko04075, 286 transcripts), “protein processing in endoplasmic reticulum” (ko04141, 278 transcripts), “starch and sucrose metabolism” (ko00500, 254 transcripts) and “RNA transport” (ko03013, 230 transcripts) (Figure 4). KEGG pathway analysis of the differentially expressed genes showed that the top several enriched KEGG pathways were environmental adaptation, signal transduction, energy metabolism and biosynthesis of other secondary metabolites (Tables S1–S5 in Supplemental file 2).

In order to characterize changes in the gene expression at a single developmental stage, transcripts that revealed differential expression between the treatments at every time point were investigated. Grape berry development and maturation are complex processes displaying a double sigmoidal growth pattern with three distinct phases [2]. The first phase involves a rapid increment of berry size and cell division, accumulations of tartrate and malate, synthesis of some precursors of phenolic compounds and procyanidins [35]. This phase had two sample times (S1 and S2). GO analysis identified 75 and 100 category enrichment, of which 57 and 61 categories had significant difference (*p* < 0.05). The main differential categories were the responses to organic nitrogen (GO:0010243), endogenous stimulus (GO:0009719), chitin (GO:0010200), water stimulus (GO:0009415), and chemical stimulus (GO:0042221) (Tables S1 and S2 in Supplemental file 1). KEGG pathway analysis revealed 143 and 50 pathways differential expression enrichment, of which 12 and seven pathways had significant difference (*p* < 0.05). The main differential pathways were plant–pathogen interaction (ko04626), plant hormone signal transduction (ko04075), nitrogen metabolism (ko00910), carotenoid biosynthesis (ko00906), stilbenoid, diarylheptanoid and gingerol biosynthesis (ko00945) (Tables S1 and S2 in Supplemental file 2). In the second phase, pigments and sugars began to accumulate, while organic acids decreased and the berries became soft [32,36]. S3 and S4 were the sample times. GO analysis identified 61 and 87 categories enrichment, of which 34 and 54 categories had significant difference (*p* < 0.05). The main differential categories were flavonoid metabolic process (GO:0009812), flavonoid biosynthetic process (GO:0009813), phenylpropanoid biosynthetic process (GO:0009699), cell wall macromolecule metabolic process (GO:0044036), and xyloglucan metabolic process (GO:0010411) (Tables S3 and S4 in Supplemental file 1). KEGG pathway analysis revealed 39 and 60 pathways with differential expression enrichment. Among them, three and seven pathways had significant difference (*p* < 0.05). The main differential pathways were flavone and flavonol biosynthesis (ko00944), flavonoid biosynthesis (ko00941), glucosinolate biosynthesis (ko00966), plant hormone signal transduction (ko04075), and circadian rhythm–plant (ko04712) (Tables S3 and S4 in Supplemental file 2). In the last phase (S5), volatile secondary metabolites including terpenes, norisoprenoids, esters and thiols were synthesized [37]. GO analysis identified 15 categories of enrichment. The main differential categories were regulation of transcription, DNA-dependent (GO:0006355), nuclear-transcribed mRNA poly(A) tail shortening (GO:0000289), negative regulation of short-day photoperiodism and flowering (GO:0048577) (Table S5 in Supplemental file 1). KEGG pathway analysis revealed three pathways of differential expression enrichment, which were protein processing in endoplasmic reticulum (ko04141), RNA degradation (ko03018), and oxidative phosphorylation (ko00190) (Table S5 in Supplemental file 2). 

In general, the data showed that root restriction could strongly impact gene expression of berries. Some functional categories with gene pathways were linked to the physical and biochemical changes throughout the sample times. This progress contained gene expression, transcriptional regulation and signal transduction at molecular level. The results improved the understanding of the regulatory networks that controlled the grape responses to RR. The transcriptomic results agreed with the biological process of relevant metabolic pathways during berry development [21].

### 2.5. Validation of Gene Expression Using qRT-PCR

To validate the accuracy and reproducibility of the expression profiles obtained by RNA-Seq, several transcripts were randomly selected for qRT-PCR. These transcripts were upregulated, downregulated and unaffected during the berry development, involved in both metabolism and biological processes. The Fragments Per Kilobase of exon model per Million mapped reads (FPKM) values, RNA-Seq and qRT-PCR fold changes are listed in Table S1 (Supplemental file 3). The qRT-PCR Fold changes in these genes were calculated. It was found that the changes generally agreed with the transcript abundance measured by RNA-Seq, showing the reliability of the RNA-Seq data.

## 3. Materials and Methods 

**Figure 5:**
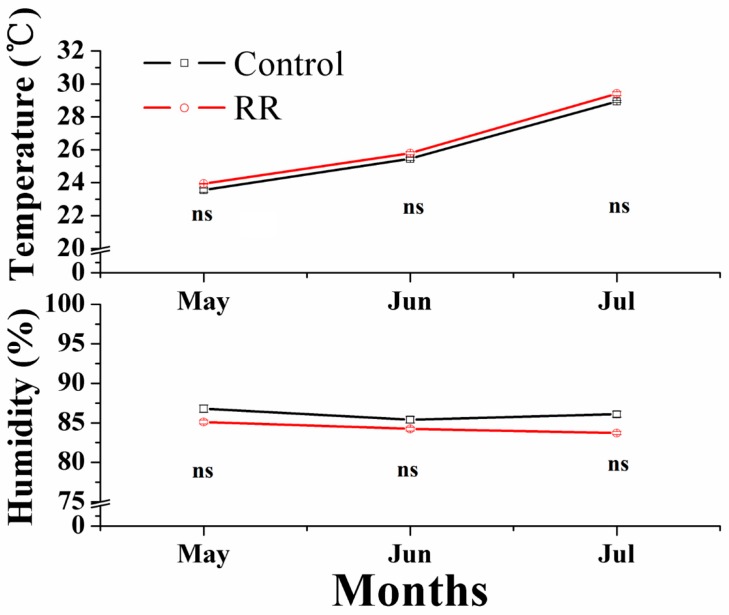
Environmental conditions in the greenhouse during berry development. RR, root restriction, ns = not significant differences (*p* > 0.05).

Two developmental series of ‘Summer Black’ table grapes under normal cultivation and RR were prepared. The RR treated grapes were planted in 40 cm depth and 100 cm wide ridges isolated by plastic film from outside ground. The control grapes were planted in raise bed (40 cm deep) with the same soil at open ground. The same watering and fertilizer strategy were applied to RR and the control plants to avoid different environmental conditions. Five fruit developmental stages, namely S1 fruitlet (15days after full bloom (DAFB)), S2 immature green (28 DAFB); S3 before veraison (42 DAFB); S4 veraison (53 DAFB) and S5 fully ripe (74 DAFB) were collected. Figure 5 shows different environmental conditions during fruit ripening. For each treatment, 10 clusters were randomly picked at each sampling time from at least 5 plants with no evidence of disease or stress symptoms. All samples were transported to the laboratory within 3 h after harvest. Berries with uniform maturity and no mechanical damage were cut into small pieces and frozen in liquid nitrogen and stored at −80 °C for future use. All treatments and controls were performed with three biological replicates.

### 3.1. Color and TSS Measurement

Fruit surface color at different ripening stages was measured by a Hunter Lab Mini Scan XE Plus colorimeter (Hunter Associates Laboratory, Inc., Reston, VA, USA). The Commission Internationale de L’Eclairage (CIE) L*a*b* color scale was adopted, and the raw data was obtained as L*, a*, b*. The CIRG, a comprehensive indicator of the color index of red grapes, was calculated according to CIRG = (180 − *H*)/(L* + C), while *C* = (a*^2^ + b*^2^)^0.5^ and *H* = arctan (b*/a*) [38,39,40]. Two measurements were made for each fruit and a mean value was obtained and set as the color of this fruit. There was a total of 30 fruits from the color measurements, and there were 15 fruits for the TSS measurement using a refractometer PR101-a (Atago, Tokyo, Japan). Each fruit had two measurements.

### 3.2. Total Soluble Phenolics, Total Anthocyanins and Total Procyanidins

Total soluble phenolics were measured using the Folin–Ciocalteu method [41] with slight modification. The lyophilized berry powder was extracted with 70% aqueous ethanol (containing 1% formic acid) in a solid to liquid ratio of 1 to 48 (m/v). The appropriately diluted extracts (0.5 mL) with 4 mL of ddH_2_O were placed in a test tube, to which 0.5 mL of 0.5 N Folin–Ciocalteu reagents was added (Sigma-Aldrich, St. Louis, MO, USA), allowed to react for 3 min, and then neutralized with 1 mL of saturated sodium carbonate. Absorbance at 760 nm was read using a spectrophotometer (DU-8000 Beckman Coultor, Fullerton, CA, USA) after 2 h incubation at 30 °C. Gallic acid was used as the standard and results were expressed as mg gallic acid equivalents/g dry weight.

Total anthocyanins were determined by the modified pH differential method [40]. The lyophilized berry powder aqueous ethanol extract prepared as previously described was diluted with 0.2 mol/L potassium chloride buffer (pH 1) or 0.2 mol/L sodium acetate buffer (pH 4.5) at a ratio of 1:4. Absorbances at 510 nm and 700 nm were measured at both pH after 20 min under darkness. Results were expressed as mg cyaniding-3-glucoside equivalents/g dry weight using a molar extinction coefficient of 29600.

Total procyanidins of extracts were measured according to the previously method [42] with slight modification. Appropriately diluted extracts (50 μL) were added to 250 μL of 4-dimethylaminocinnamaldehyde solution (hydrochloric acid and ethanol; 1:9 *v*/*v*) to initiate the reaction. After 15 min, absorbance at 640 nm was recorded using a microplate reader (Thermo, Electro Co., Waltham, MA, USA). The results were expressed as mg procyanidin B2 equivalents/g dry weight.

### 3.3. RNA Extraction and RNA-Seq

Total RNA was extracted from some frozen whole grape berry powder according to our previously published method [43]. After removal of contaminating genomic DNA with a TURBO DNA-free kit (Sigma-Aldrich, St. Louis, MO, USA), the total RNA was quantified using Nanophotometer Pearl (Implen, Germany), and used for RNA-seq and real-time PCR. All of the samples were performed with three biological replicates. For RNA-Seq, the cDNA libraries were constructed using the TruSeq^TM^ RNA Sample Preparation Kit (Illumina, Inc., San Diego, CA, USA) for each exocarp, and the raw read sequences were obtained by the Shanghai Majorbio Bio-pharm Biotechnology Co. (Shanghai, China) using Illumina HiSeqTM 2000 with 5 Gb reads per sample. The raw reads were initially processed to get clean reads by removing the adapter and low quality sequences using the software SeqPrep (https://github.com/jstjohn/SeqPrep). The clean reads were aligned to the reference *Vitis vinifera* genome (http://www.genoscope.cns.fr/externe/Download/Projets/Projet_ML/data/) [15] using TopHat 2.0.13 software (http://tophat.cbcb.umd.edu/) [44] and the quality was assessed by saturation analysis, duplicate reads analysis and gene coverage analysis using RSeQC-2.3.2 program (http://code.google.com/p/rseqc/) [45]. Gene expression values were calculated and correlation analysis by the read/fragments per kilobase of exon per million fragments mapped reads (RPKM/FPKM) using the Cuffdiff 2.2.1 program (http://cufflinks.cbcb.umd.edu/). Differential expression was analyzed according to the count values of each transcript in two libraries using edgeR 3.16.0 software (http://www.bioconductor.org/packages/release/bioc/html/edgeR.html). Genes with a false discovery rate (FDR) < 0.05 and estimated absolute log_2_ fold change (FC) > 1 were used as the thresholds for judging significant difference in transcript expression [46,47,48]. Differentially expressed genes were identified using the software of VennDiagram 1.6.7 (http://en.wikipedia.org/wiki/Venn_diagram) [49] by running the R 3.0.1 program (https://www.r-project.org/). Gene ontology (GO, http://www.geneontology.org) terms of transcripts were identified and annotated by the blast2go pro software http://www.blast2go.com/b2ghome) [50] (data are in Supplemental file 4). GO functional enrichment analysis was performed based on goatools 0.5.9 https://github.com/tanghaibao/goatools) [51]. KEGG (http://www.genome.jp/kegg/) pathway analysis was performed using the KEGG function of the blast2go webtool. KEGG pathway analysis of the differentially expressed genes was performed using KOBAS 2.0 http://kobas.cbi.pku.edu.cn/home.do) [52].

All of these RNA-Seq reads were deposited in NCBI SRA (Sequence Read Archive) (https://www.ncbi.nlm.nih.gov/sra). The accession codes are: SRX2234711/SRR4408346, SRX2234711/SRR4408347, SRX2234711/SRR4408413, SRX2234711/ SRR4408414. 

### 3.4. qRT-PCR Validation of RNA-Seq Data

For qRT-PCR analyses, gene-specific oligonucleotide primers were designed and described in Table 3. The gene specificity of each pair of primers was checked by melting curves and product re-sequencing twice. The GAPDH gene was employed as the internal control for calculating relative expression of the mRNA [53]. The sequences of GAPDH primers are described in Table 3. Real-time PCR was performed using FastStart Universal SYBR Green (Roche, Basel, Switzerland), initiated by 10 min at 95 °C and followed by 40 cycles of 95 °C for 30 s, 60 °C for 30 s, and then by 72 °C for 10 min, and completed with a melting curve analysis program. The PCR mixture (10 μL total volume) was comprised of 5 μL of Roche FastStart Universal SYBR Green Master (ROX) (Roche, Basel, Switzerland), 0.75 μL of each primer (10 μM), 0.5 μL of diluted cDNA and 3 μL of PCR-grade ddH_2_O. No-template controls and melting curve analysis were included for each gene during each run.

### 3.5. Statistical Analysis

The statistical significance of differences was calculated by ANOVA. The results are the mean ± SE of at least three independent replicates and were analyzed using data processing system SPSS16.0 statistical software package (Chicago, IL, USA). Figures were drawn by Origin 8.0 (Microcal Software Inc., Northampton, MA, USA).

## 4. Conclusions

On the basis of RNA-Seq analysis of transcriptomes, this study implemented a global investigation of differential gene expressions triggered by RR treatment during berry development. It was found that RR was able to increase TSS and alter anthocyanin biosynthesis. The contents of TSS, total phenolics, total anthocyanins and total procyanidins were closely correlated with genes involved in their functional categories and biosynthesis/degradation. This was the first report showing that RR had significant effects on transcriptomes of grape berries.

## Figures and Tables

**Table 1:** Summary of sequencing data and statistics of the transcriptome assembly during developmental stages.

Statistical Analysis	Treatments	Developmental Stages				
		S1	S2	S3	S4	S5
Raw bases (bp)	Control	6043206288	6388028052	5629860684	5937329496	6421364544
	RR	5863704000	6208024116	5503911840	5821091052	5730388524
Raw reads (No.)	Control	47961955	50698635	44681434	47121663	50963211
	RR	46537333	49270033	43681840	46199135	45479274
Clean bases (bp)	Control	5507614346	5804699908	5088328621	5360779171	5781234462
	RR	5326390300	5642227230	4920575938	5245451851	5189109353
Clean reads (No.)	Control	44822587	47465685	41663959	44128070	47813283
	RR	43519598	45949313	40551247	43479523	42667291
≥Q30	Control	93.62	93.31	93.22	93.11	92.91
	RR	94.09	93.76	92.81	93.08	93.12
Mapped sequences (No.)	Control	33064934	31668177	27537423	30466585	31557453
	RR	31883094	28392274	29022488	25252114	26946449
Mapped percentage (%)	Control	73.74	66.94	66.27	68.99	67.01
	RR	73.34	61.45	71.51	59.55	63.06

**Table 2:** Numbers of differentially expressed genes (FDR < 0.05 and Log_2_FC > 1) during developmental stages under root restriction treatment. FDR, false discovery rate; FC, fold change.

Log_2_FC	Upregulated Genes	Downregulated Genes	Not Differentially Expressed
RR_S1_/Control_S1_	987	277	28707
RR_S2_/Control_S2_	72	241	29658
RR_S3_/Control_S3_	112	29	29830
RR_S4_/Control_S4_	158	88	29725
RR_S5_/Control_S5_	11	8	29952

**Table 3:** Primers for qRT-PCR.

Gene	Forward Primer (5′ to 3′)	Reverse Primer (5′ to 3′)
GAPDH	TGGAGCTGAATTTGTTGT	GTGGAGTTCTGGCTTGTA
VIT_04s0023g02290	TTTGTTTGCGGTCTTGGA	GAACAGCCTGCCGTAGAA
VIT_05s0049g00770	CCACCATCTCCCACCCAT	TGTCACAATACTCATCACCC
VIT_07s0197g00240	AGCCATTTATCAGAGCGAACAG	GCACCAGCTTGAGGAGAACAT
VIT_09s0002g06590	ATGAATACAACTTCGTCCTT	GCTTTGAGTTCAGCCATT
VIT_14s0068g00920	TCCCAGGGTTGATTTCCA	TGCTGCCTTTCCCTTCTT

## Supplementary Materials

Supplementary materials can be accessed at: http://www.mdpi.com/1420-3049/21/11/1431/s1.
